# Stratified Mucin-Producing Intraepithelial Lesion (SMILE) of the Uterine Cervix: High-Risk HPV Genotype Predominance and p40 Immunophenotype

**DOI:** 10.3390/cells10082039

**Published:** 2021-08-10

**Authors:** Margareta Strojan Fležar, Neža Nedelko, Mario Poljak, Anja Oštrbenk Valenčak, Helena Gutnik

**Affiliations:** 1 Institute of Pathology, Faculty of Medicine, University of Ljubljana, Korytkova 2, 1000 Ljubljana, Slovenia; neza.nedelkox3@gmail.com; 2 Institute of Microbiology and Immunology, Faculty of Medicine, University of Ljubljana, Zaloška Cesta 4, 1000 Ljubljana, Slovenia; mario.poljak@mf.uni-lj.si (M.P.); anja.osterbenk@mf.uni-lj.si (A.O.V.)

**Keywords:** cervical precancerous lesion, stratified mucin-producing intraepithelial lesions, HPV genotyping, immunohistochemistry, high-risk HPV types, HPV genotyping

## Abstract

Stratified mucin-producing intraepithelial lesion (SMILE) is a rare high-grade cervical precancerous lesion designated a variant of adenocarcinoma in situ (AIS) in the WHO classification. We aimed to determine HPV genotypes, immunohistochemical phenotype and mucin presence in SMILE. Between 2010 and 2018, SMILE was diagnosed in 34 out of 6958 (0.5%) cervical biopsies, in 23 patients. Twenty-six tissue samples from twenty-one patients were available for further analysis, including 13 with SMILE alone, 12 with SIL and/or AIS and one with HSIL, AIS and endocervical adenocarcinoma. HPV genotyping was performed using the Seegene Anyplex II HPV 28 assay. Of the 26 samples, a single HPV genotype was identified in the majority of cases (*n* = 22), including 12/13 SMILEs associated with SIL/AIS. All but one were high-risk HPV genotypes (23/24; 96.8%). We identified seven different HPV genotypes, the most common being HPV16 (*n* = 10; 43.5%), HPV18 (*n* = 8, 34.8%) and HPV 31 (*n* = 5, 21.7%). All SMILEs showed a strong positive reaction to p16, CK7, CK19 and high Ki67 expression comparable to adjacent HSIL and/or AIS if present. SMILE showed variable mucin presence and p40-positive squamous differentiation suggesting phenotypic diversity in cervical precancerous lesions infected by single HPV.

## 1. Introduction

Stratified mucin-producing intraepithelial lesion (SMILE) is a rare cervical precancerous lesion exhibiting architectural features encompassing cellular stratification similar to squamous intraepithelial lesions (SIL), crowding, an increased nuclear/cytoplasmic ratio, hyperchromasia, mitoses and apoptosis. Additionally, cytoplasmic mucin is found in variable proportions of cells diffusely through all epithelial layers, showing as cytoplasmic vacuoles or, alternatively, as cytoplasmic clearing with increased distance between the nuclei, without classical gland formation [1,2]. In the 2014 World Health Organization (WHO) and recently in the 2020 WHO classification of cervical tumors, SMILE has been categorized as a subtype of endocervical adenocarcinoma in situ (AIS), while initial and later reports acknowledged a double histomorphological differentiation, namely, glandular and squamous [1,2,3,4].

In the majority of cases reported, SMILE was associated with conventional high-grade squamous intraepithelial lesions (HSIL), adenocarcinoma in situ (AIS) or both [1,2,5,6,7,8]. Moreover, many reported cases also contained an invasive component of squamous carcinoma, adenocarcinoma or adenosquamous carcinoma [1,2,5]. SMILE has also recently been described as a precursor of invasive stratified mucin-producing carcinoma [9].

By analogy to squamous intraepithelial lesions (SIL) and AIS, SMILE has been considered etiologically linked to persistent infection with high-risk human papillomavirus (HPV) genotypes, mostly due to strong diffuse block p16 nuclear and cytoplasmic positivity, although HPV genotyping has only been reported in a very small number of studies on a limited number of cases [5,6,8,10].

SMILE presumably originates from reserve cells of the transformation zone (TC), which are capable of multidirectional differentiation [1,2]. Cytokeratin (CK) 19 and CK7 pair in simple epithelia and both stain reserve cells. CK19 is also expressed in basal layers of ectocervical squamous epithelium [11,12]. Moreover, CK7 has been included among the biomarkers of squamocolumnar junction (SCJ) cells shown to be related to carcinogenic HPV-associated cervical neoplasia [13,14]. Both CK7 and CK19 have also been detected in the majority of HSIL and cervical carcinomas, and suggested to be used as biomarkers of integration of high-risk HPV in cervical lesions [11,12,13].

Mucinous differentiation in SMILE has been highlighted by Alcian Blue staining in some studies, while immunohistochemical staining for p63 was used in seminal studies to assess squamous differentiation [1,2]. p40 or ΔNp63 is one of ten isoforms of protein p63 and is considered to be the key regulator of myoepithelial, squamous and basal cell differentiation [2,15,16]. Compared to the p63 antibody, p40 is a more specific biomarker of squamous differentiation, and the immunohistochemical staining of p40 is used for the detection of squamous differentiation and the exclusion of glandular and neuroendocrine differentiation in cervical carcinomas [17,18].

The aim of our study was to assess the distribution of HPV genotypes in SMILE, together with p16, Ki 67, CK7 and CK19 immunohistochemical staining to evaluate the integration of HPV and putative derivation from SCJ and/or reserve cells. The epithelial differentiation of SMILE was assessed by the immunohistochemical staining of p40 for putative squamous differentiation and by Alcian blue for mucin content reflecting glandular differentiation, and to compare it to adjacent SIL and/or AIS where present.

## 2. Materials and Methods

We retrieved from the electronic archive of the Institute of Pathology, Faculty of Medicine, University of Ljubljana, all cases of cervical lesions diagnosed as SMILE between 2010 and 2018. Of the 6958 cervical tissue biopsies analyzed, 34 (0.5%) biopsies from 23 patients were diagnosed as SMILE (by MSF, HG) according to histomorphological criteria published by Park et al. [1]. Twenty-six samples from 21 patients (16 had one biopsy, 5 had 2 biopsies) were included in the final analyses. All biopsies were reviewed and assessed for SMILE, SIL, AIS, carcinoma, non-neoplastic squamous, squamous metaplastic and endocervical glandular epithelium (by MSF, HG).

For HPV genotyping, three 10 μm thick sections were cut from each tissue block and processed for DNA extraction following our standard laboratory protocol, as described in detail previously [19]. Briefly, tissue sections were incubated with 180 μL of buffer ATL and 20 μL of proteinase K overnight, on a rocking platform at 56 °C and 400 rpm, followed by incubation at 90 °C for 1 h and DNA extraction using a QIAamp MinElute Media Kit (Qiagen, Hilden, Germany) following the manufacturer’s instructions. Bound DNA was finally eluted with 100 µL of Buffer AE and quantified using a Qubit fluorometer (Thermo Fisher Scientific, Waltham, MA, USA). All precautions were taken to prevent possible sample-to-sample contamination, as described in detail previously [19]. All samples were tested with the widely used HPV genotyping test Anyplex HPV 28 (Seegene, Seoul, Korea), which is capable of recognizing 28 HPV genotypes: HPV6, 11, 16, 18, 26, 31, 33, 35, 39, 40, 42, 43, 44, 45, 51, 52, 53, 54, 56, 58, 59, 61, 66, 68, 69, 70, 73, and 82.

Immunohistochemical staining for p16, Ki67, CK7, CK19 and p40 was performed using protocols summarized in Table 1. Alcian Blue special staining was used for the detection of mucin (Alcian Blue Staining Kit, Ventana Roche, Tucson, AZ, USA).

Immunohistochemical stains and Alcian blue staining were independently assessed by two certified pathologists (MSF, HG). In discrepant cases, slides were reviewed on a multihead microscope to achieve consensus. Immunoreaction to p16 was considered positive when diffuse block nuclear and cytoplasmic positivity was observed. For squamous lesions and SMILE, Ki-67 increased expression was assessed as nuclear positivity found in the lower third, lower two-thirds and in more than two-thirds of epithelial layers, with parabasal positivity considered normal, while increased immunoreactivity was assessed for AIS. For CK7 and CK19, cytoplasmic positivity was assessed; for p40, nuclear immunoreaction was considered positive. A blue cytoplasmic reaction confirmed mucin presence by Alcian Blue staining.

Statistical analyses were performed with Statistical Package for Social Sciences (SPSS). The Mann–Whitney U test was used to compare the staining percentage of different immunohistochemical biomarkers between groups and in the case of categorical variables, the χ^2^ test was used. Differences in the distribution of HPV genotypes between SMILE and SMILE combined with SIL and/or AIS were calculated with Fisher’s exact test. Reported *p* values ≤ 0.05 were considered to be statistically significant.

The study was approved by the Slovenian National Medical Ethics Committee (consent No. 0120-526/2018/5).

## 3. Results

Twenty-six tissue samples with SMILE from 21 patients, with a median age of 33 years (range: 20–49 years), were used in this study. Sixteen out of twenty-six (61.5%) were small cervical biopsies and 10/26 (38.5%) were cone excision biopsies (3 by scalpel, 7 by LLETZ). In 13/26 (50%) tissues biopsies (3 cones excisions, 10 small biopsies), SMILE was combined with other precancerous lesions (Table 2). One SMILE combined with both HSIL and AIS was associated with an endocervical adenocarcinoma of the uterine cervix, FIGO stage IA1, but was no longer present in deeper tissue sections for adjunct studies.

As shown in Table 2, HPV was detected in 24/26 (92.3%) SMILE samples, but not in two samples, possibly due to the nature of the archived specimens (partial DNA degradation). However, when we compared both Qubit reading values as well as Ct values of Anyplex HPV 28 internal control, no apparent difference was recorded between 24 HPV-positive and 2 HPV-negative samples for either value. In 22/26 (91.7%) samples, a single HPV genotype was identified, in one sample two HPV genotypes and in one sample three HPV genotypes. All HPV genotypes but one belonged to high-risk HPVs (23/24, 95.8%). In total, we identified six different high-risk HPV genotypes (HPV16, 18, 31, 33, 39, and 51), with HPV16 being the most common (10/23 cases, 43.5%), followed by HPV18 (8/23 cases, 34.8%) and HPV31 (5/23 cases, 21.7%). A single HPV genotype was found in the majority of combined lesions (12/13, 92.3%), except for one SMILE with AIS, in which two genotypes, HPV 16 and 18, were found. A lesion with three HPV genotypes, HPV31, 39 and 51, was SMILE only. A possibly carcinogenic HPV70 genotype was detected in one sample with SMILE only. Statistical analysis showed that HPV16 and HPV18 were more frequent in SMILE combined with other precancerous lesions (*p* = 0.0233).

**Figure 1:**
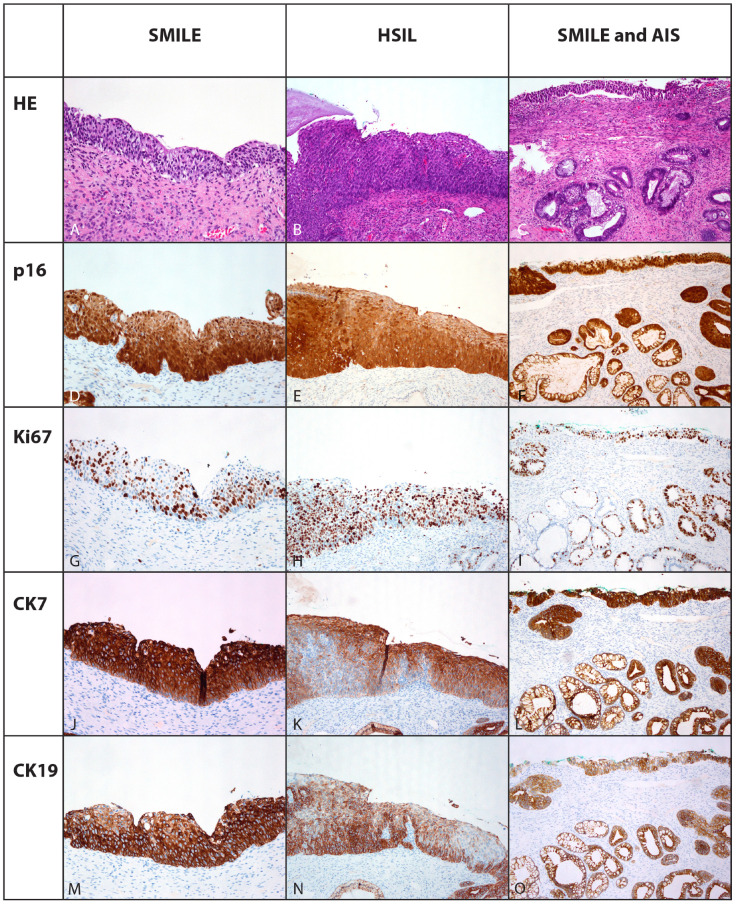
Immunohistochemical expression of p16, Ki67, CK7 and CK19 in stratified mucin-producing intraepithelial lesion involving the surface epithelium (**A**,**D**,**G**,**J**,**M**), in high-grade intraepithelial squamous lesion, focally underlaying endocevical epithelium (**B**,**E**,**H**,**K**,**N**) and endocervical adenocarcinoma in situ in the endocervical crypts with remnants of SMILE in the surface epithelium (**C**,**F**,**I**,**L**,**O**).

Diffuse strong nuclear and cytoplasmic block positivity to p16 was found in all cases of SMILE, LSIL, HSIL and AIS, although it was confined to the lower two-thirds of the epithelium in LSIL compared to the whole thickness in other lesions (Table 3, Figure 1).

**Figure 2:**
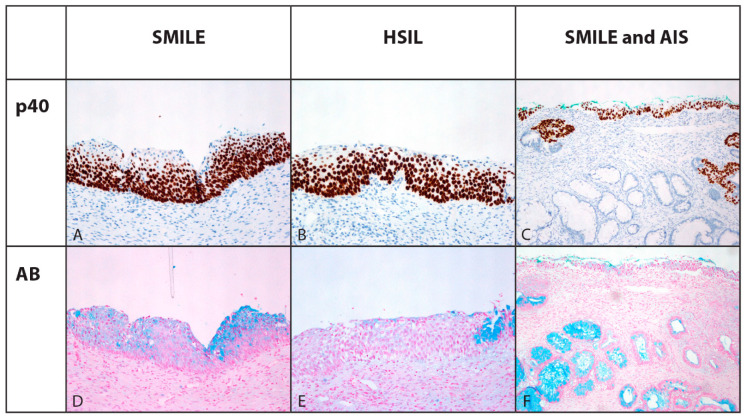
Immunohistochemical expression of biomarker p40 in lower two-thirds (**A**) and presence of mucin by Alcian blue staining throughout the full thickness (**D**) of stratified mucin-producing intraepithelial lesion (×200). Similar expression of p40 in adjacent high-grade squamous intraepithelial lesion (**B**) with some mucin in the remaining overlaying endocervical epithelial cells focally (**E**). Predominant lack of p40 expression (**C**) and presence of mucin (**F**) in endocervical adenocarcinoma in situ; however, focal p40 positivity can be seen in the epithelial islands in the upper right and left quadrant of the specimen, and p40 positivity and scattered mucin vacuoles can be seen in the remnants of the SMILE in the surface epithelium.

Parabasal cell nuclear positivity to Ki-67 was observed in 16/17 (94.1%) cases in non-neoplastic squamous or squamous metaplastic epithelium, while rare single cells were positive in endocervical glandular (16/26, 61.5%) and reserve cells (5/14, 35.7%). SMILE exhibited increased nuclear positivity in all cases (Table 3, Figure 2). However, it was confined to the lower third of the lesion in two (7.7%), to the lower two-thirds in seven (26.9%) and present throughout the whole thickness in 17 cases (65.4%) (Table 3, Figure 1).

CK7 was not detected in six (35.3%) cases in the areas of non-neoplastic squamous and squamous metaplastic epithelium and in LSIL, while in eight (47.1%) cases the areas of non-neoplastic squamous and squamous metaplastic epithelium exhibited less than 10% of positive cells, one (5.9%) case 10 to 50% and two (11.7%) cases more than 50% of positive cells (Table 3). All cases with endocervical glandular epithelium, reserve cells, SMILE, AIS and 4/8 (50%) of HSIL exhibited diffuse positivity for CK7, while the other half of HSIL exhibited uneven patchy positivity; however, more than 50% of cells were CK7-positive (Table 3, Figure 1).

The expected basal positivity for CK19 was observed within non-neoplastic squamous and squamous metaplastic epithelium in the majority of cases (11/17, 64.7%). In the remaining cases, CK19 was positive in 10–50% of cells in 4/17 (23.5%) cases and in 10% of cells in one case (5.9%). More than 50% of cells were positive in all cases, within endocervical glandular epithelium, reserve cells, SMILE, AIS, HSIL. CK19 was negative in LSIL and in one case of non-neoplastic squamous and squamous metaplastic epithelium (Table 3, Figure 1).

In 13/17 cases (76.5%) of non-neoplastic squamous and squamous metaplastic epithelium, as well as in 6/26 (24.1%) SMILE and LSIL, p40 immunopositivity was confined to the lower two-thirds of the epithelium, while it was found in the whole thickness of the majority (20 cases, 78.9%) of SMILEs and in all HSIL (Figure 2). Focal p40 nuclear positivity was observed in two out of five AIS, staining up to 15% of basal cells (Table 3).

Alcian blue staining showed mucin in endocervical glandular epithelium, SMILE and AIS, although the percentage of cells showing mucin was lower in SMILE than in AIS (median 20%, range 10–37.5% vs. median 30%, range 20–70%, *p* = 0.21/NS) (Table 3, Figure 2). Scattered single cells with mucin were present in the superficial layer in one HSIL (Figure 2).

## 4. Discussion

Glandular features with the presence of cytoplasmic mucin together with stratified architecture resembling SIL have been described for SMILE, which was included in the category of endocervical AIS for the first time in the 2014 edition, and again in the 2020 edition, of the WHO classification of female genital tumors [1,3,4].

Reliable data on the epidemiology of SMILE are lacking, although it has been reported to be a very rare cervical precancerous lesion. In our study, SMILE was diagnosed in the original histopathology reports and confirmed on the review (by MSF and HG) in 0.5% of cervical biopsies identified in our institutional computer files, a number similar to the previously reported 0.6% in a study in which all reports with endocervical AIS were reviewed to ascertain the presence of SMILE [2]. In a study by Onishi et al., the incidence of SMILE was higher, 2.7%, possibly because they only included HSILs and carcinomas of cervical cone excision biopsies and hysterectomy specimens [5]. The incidence of SMILE has not been reported in other studies [1,6,7]. In accordance with the comments in previous reports of SMILE, the incidence might be underestimated, especially if pathologists were not familiar with the entity [2,3,4].

In this study, SMILE coexisted with squamous and glandular high-grade intraepithelial lesions in 50% of cases, while previous studies have reported a majority of lesions associated with HSIL and/or AIS and/or carcinoma, namely, 92.3% to 100% [2,5,6,7]. SMILEs in our study were more frequently associated with HSIL (six cases, 23.1%) than with AIS (four cases, 15%), while all three lesions were found in two cases (5.5%). Our findings are consistent with previous data reporting SMILE more often associated with HSIL (ranging from 76.4% to 93%) [1,2,6,7] compared to AIS (ranging from 42% to 62%), while it was more strongly associated with AIS (in 92%) only in a single study [5]. Only a few studies have specifically reported data on mixed lesions, which were found in 20.6% to 46% of cases [5,6,7]. We found one SMILE associated with LSIL/CIN1, which seems to be an exceptional finding, reported only in a single previous study [2]. In contrast to other reports, only one case of SMILE in our study was associated with an adenocarcinoma of the uterine cervix, FIGO stage IA1 together with AIS and HSIL. All the lesions in our study were detected in asymptomatic women enrolled in the national cervical screening program (ZORA), which may explain the low incidence of carcinoma associated with SMILE [20]. Other histopathology studies have found associated carcinomas of different histological types in 10% to 100% of cases [1,2,5,6,7]. Studies reporting invasive stratified mucin-producing carcinoma have been evolving recently, suggesting that SMILE is a distinct precancerous lesion related to this specific histologic type of cervical carcinoma, which was included as a separate entity among endocervical adenocarcinomas in the last WHO classification [9,21].

Similar to previous studies, all SMILEs in this study exhibited strong block diffuse nuclear and cytoplasmic positive p16 immunostaining consistent with transforming HPV infection [1,2,5,8,9,10]. Except in one SMILE, when only a possibly carcinogenic HPV70 genotype was identified, high-risk HPV genotypes were detected in all other SMILEs.

In the majority of lesions (22/24, 91.7% cases), only one HPV genotype was identified, including cases in which SMILE was combined with HSIL and/or AIS (12/13, 92.3%). It is noteworthy that a single HPV genotype was detected in cervical tissue biopsies encompassing SMILE with SIL and/or AIS. It has previously been recognized by laser-capture microdissection studies of cervical tissue with multiple HPV genotypes detected that a single HPV genotype could be mapped to a specific tissue location and identified as responsible for the neoplastic process, whereas additional HPV genotypes were detected in uninvolved mucosal regions or in cellular debris, a phenomenon described as “one virus, one lesion” [22]. Our study shows that, conversely, a single HPV genotype can account for histologically different lesions evolving from the cervical transformation zone or, more probably, originating from the same SCJ cells [13].

In one previous report, individual HPV16 and HPV18 were detected in four and seven SMILE cases, respectively, using the Roche COBAS 4800 HPV test, while four cases were positive with a pool of 12 other high-risk HPV genotypes (HPV 31, 33, 35, 39, 45, 51, 52, 56, 58, 59, 66 and 68) [6]. Another study showed nuclear signals by HPV DNA in situ hybridization (ISH) (pooled subtypes 16, 18, 31, 33, and 51) in all 12 cases of SMILEs, but they could detect HPV18 and HPV16 by E7 PCR products in only 50% of cases, namely, HPV18 in five and HPV16 in a single case [5]. Among published SMILE case reports, one case was positive for HPV16 and another for HPV18 by multiplex PCR and ISH with an HPV16/18 probe [8]. Another case was positive for HPV52 and 68 by multiplex PCR and subsequently with RNA fluorescent ISH [10]. Compared to other studies, we found HPV16-positive SMILE cases more frequently, although both HPV16 and HPV18 were predominant genotypes, similar to other reports. We found that HPV16 and HPV18 were more frequent in SMILE combined with other precancerous lesions, which has not been shown previously. The most plausible explanation for this finding could be the fact that among the combined lesions included in our study, all but one were HSIL+. Because HPV16 is the most frequent HPV genotype worldwide associated with severe cervical precancerous lesions, hence the borderline significant difference (*p* = 0.0233) in HPV16 and HPV18 frequency between SMILE only vs. SMILE combined with other precancerous lesions. In this study, we also showed that HPV31 occurred in SMILE either alone or combined with other HPV genotypes, a finding similar to those of other HSIL lesions. Moreover, one SMILE was found to be associated with possibly carcinogenic HPV, namely, HPV70, which has not been reported previously.

In accordance with the transforming HPV infection indicated by diffuse p16 positivity in all SMILE cases, the proliferation activity of cells was also increased, as detected by Ki67 immunostaining, and the results are consistent with previous reports [1,2,10]. Ki67 expression was limited to the lower two-thirds of epithelial thickness in about one-third of cases, which may nonetheless reflect lesions with a lesser grade of intraepithelial neoplastic changes.

Expression of CK7 and CK19 by immunohistochemical staining was found in AIS and/or HSIL, as expected according to previous reports, and likewise in all SMILEs, which has not been previously demonstrated [11,12,13,14]. Additionally, CK7 was listed among biomarkers of SCJ cells shown to be related to carcinogenic HPV-associated cervical neoplasia, and both CK7 and CK19 were found to stain reserve cells of the uterine cervix in earlier studies [11,13]. Expression of CK7 in conjunction with CK19 in SMILE would support an origin of the lesion from SCJ cells and/or reserve cells, on the one hand, and an indication of high-risk HPV integration also concordant with diffuse block p16 expression, on the other [12,13,23].

This study showed positive immunohistochemical staining for p40 (ΔNp63) in SMILE; p40 is one of the many isoforms of p63 and is considered to also be a biomarker of the squamous lineage in other tumor sites, especially in diagnosing and triaging lung carcinoma for therapy-related molecular-genetic and immunotherapy studies [15,16,17,24]. In the majority of SMILE lesions in this study (20 cases, 78.9%), p40 positivity was found in the whole thickness of the lesion, an observation similar to that of adjacent HSIL where present, although it was confined to the lower two-thirds of the epithelium in 6/26 (24.1%) SMILE and in LSIL. Unexpected focal p40 nuclear positivity was observed in 2/5 AIS with conventional histomorphological features, staining up to 15% of basal cells, possibly representing residual reserve cells, which also stained strongly to p40 in all other cases (Figure 2). Dual p40 immunohistochemical staining and Alcian blue staining was described in two case reports of SMILE and p40 was positive in many cells but not in goblet cells [8]. However, no larger series have reported results of p40 immunostaining in SMILE. Previous studies used p63 immunostaining to detect squamous differentiation in SMILE and reported absent staining or positivity sharply reduced to basal cells compared to normal or neoplastic squamous epithelium, which was interpreted as consistent with columnar differentiation [1]. Onishi et al. also reported negative or only focally and weakly positive p63 and CK5/6 staining in their SMILE series [5]. Based on p63 negativity or basal p63 positivity, they postulated that p63-positive cells were either immature squamous cells undergoing endocervical cell differentiation or they were indifferent neoplastic reserve cells undergoing a wide range of differentiation types associated with SMILE described as phenotypic instability [1]. Based on the positive finding in all SMILEs in this study using p40 immunostaining, the dual adeno- and squamous differentiation of SMILE could be reconsidered. Moreover, the combination of SMILE with SIL and/or AIS in the same tissue biopsy also observed in all previous studies indicates a phenotypic diversity of precancerous lesions of the cervical transformation zone infected with a single HPV genotype, which might be overlooked or ignored in the routine diagnostic assessment of cervical biopsies.

In relation to glandular differentiation as a hallmark of SMILE, and mucin presence shown by Alcian blue, the proportion of cells with mucinous vacuoles throughout all epithelial layers varied between lesions, not only in this study but also in the original and subsequent studies [1,2]. Variable mucin expression could reflect the phenotypic diversity of cervical neoplasia also observed in the development of SMILE, with variable levels of dual differentiation, possibly squamous and glandular.

In conclusion, SMILE was found to be associated with different histological types and grades of precancerous lesions and carcinoma, similar to previous studies, although we found a lower proportion of lesions combined with HSIL and/or AIS and only one case of a small endocervical adenocarcinoma, possibly since the study was performed on women attending the organized population-based cervical cancer screening program resulting in low cervical cancer burden. We found a predominance of common high-risk HPV genotypes, namely, HPV16, 18 and 31, generally associated with high-grade cervical precancerous lesions. Our study also showed that a single HPV genotype can account for histomorphologically different lesions evolving from the cervical transformation zone, namely, SMILE in combination with HSIL and/or AIS, and that the lesions probably originate from the same SCJ cells. Moreover, we found ubiquitous p40 expression pointing to squamous along with glandular differentiation in SMILE and suggesting phenotypic diversity in cervical precancerous lesions.

## Figures and Tables

**Table 1:** Immunohistochemical protocols and antibodies used for the detection of p16, Ki67, CK7, CK19 and p40.

Antibody	Clone	Dilution	Vendor	Instrument	Detection System
p16	CINtec^®^ Histology V-Kit	RTU *	Ventana Roche	Benchmark ULTRA	OptiView
Ki-67	MIB-1	1:50	Dako	Benchmark XT	Ultraview
CK7	OV-TL 12/30	1:100	Dako	Benchmark XT	Ultraview
CK19	RCK 108	1:20	Dako	Benchmark XT	Ultraview
p40	BC28	RTU *	Ventana Roche	Benchmark ULTRA	OptiView

* RTU—Ready To Use.

**Table 2:** Combination of SMILE with other cervical precancerous lesions and results of HPV genotyping.

Diagnosis	N (%)	HPV Genotype (N)							
		HPV16	HPV18	HPV 16/18	HPV31	HPV33	HPV31/39/51	HPV70	ND
SMILE	13 (50)	2	3	0	3	1	1	1	2
SMILE and LSIL	1 (3.8)	1	0	0	0	0	0	0	0
SMILE and HSIL	6 (23.1)	3	2	0	1	0	0	0	0
SMILE and AIS	4 (15.4)	2	1	1	0	0	0	0	0
SMILE, HSIL and AIS	2 (7.7)	1	1	0	0	0	0	0	0
Total N (%)	26 (100)	9 (34.6)	7 (26.9)	1 (3.8)	4 (15.3)	1 (3.8)	1 (3.8)	1 (3.8)	2 (7.7)

**Table 3:** Results of immunohistochemical staining for p16 (diffuse block nuclear and cytoplasmic positivity), Ki-67 (increased expression), CK7 (cytoplasmic positivity in >50% cells), CK19 (cytoplasmic positivity above basal layer in >50% cells), p40 (nuclear positivity) and staining for mucin by Alcian blue.

Diagnosis	N	p16	Ki-67	CK7	CK19	p40	Alcian Blue
Squamous/metaplastic epithelium	17	0 (100%)	1 (5.9%)	11 (64.7%)	5 (29.4%)	17 (100%)	0 (0%)
Endocervical glandular epithelium	26	0 (0%)	0 (0%)	26 (100%)	26 (100%)	0 (0%)	26 (100%)
Reserve cells	14	0 (0%)	0 (0%)	14 (100%)	14 (100%)	14 (100%)	0 (0%)
SMILE	26	26 (100%)	26 (100%)	26 (100%)	26 (100%)	26 (100%)	26 (100%)
AIS	6	6 (100%)	4 (66.6%)	6 (100%)	6 (100%)	2 (40%)	5 * (100%)
LSIL	1	1 (100%)	1 (100%)	0 (0%)	0 (0%)	1 (100%)	0 (0%)
HSIL	8	8 (100%)	8 (100%)	8 (100%)	8 (100%)	8 (100%)	1 (12.5%) **

N: number of cases, * AIS was cut out in deeper tissue sections in one case, ** Positive only in the superficial layer of SIL.

## Data Availability

The data presented in this study are available on request from the corresponding author.

## References

[1] Park J.J., Sun D., Quade B.J., Flynn C., Sheets E.E., Yang A., McKeon F., Crum C.P. (2000). Stratified Mucin-Producing Intraepithelial Lesions of the Cervix. Adenosquamous or Columnar Cell Neoplasia?. Am. J. Surg. Pathol..

[2] Boyle D.P., McCluggage G.W. (2015). Stratified mucin-producing intraepithelial lesion (SMILE): Report of a case series with associated pathological findings. Histopathology.

[3] Wilbur D.C., Colgan T.J., Ferenczy A.S., Hirschowitz L., Loening T., Mc_Cluggage W.G., Mikami Y., Park K.J., Ronnett B.M., Schneider A., Kurman R.J., Carcangiu M.L., Herrinngton S., Young R.H. (2014). Glandular tumors and precursors. Tumours of the uterine cervix. WHO Classification of Tumors of Female Reproductive Organs.

[4] Crum C.P., Hoang L.N., Kong C.S., Park K.J., Parra-Herran C., WHO Classification of Tumours Editorial Board (2020). Adenocarcinoma in situ, HPV-associated, of the uterine cervix. WHO Classification of Tumours. Female Genital Tumours.

[5] Onishi J., Sato Y., Sawaguchi A., Yamashita A., Maekawa K., Sameshima H., Asada Y. (2016). Stratified mucin-producing intraepithelial lesion with invasive carcinoma: 12 cases with immunohistochemical and utrastructural findings. Hum. Pathol..

[6] Schwock J., Ko H.M., Dubé V., Rouzbahman M., Cesari M., Ghorab Z., Geddie W.R. (2016). Stratified mucin-producing intraepithelial lesion of the cervix: Subtle features not to be missed. Acta Cytol..

[7] Backhouse A., Stewart C.J.R., Koay M.H.E., Hunter A., Tran H., Farrell L., Ruba S. (2016). Cytologic findings in stratified mucin-producing intraepithelial lesion of the cervix: A report of 34 cases. Diagn. Cytopathol..

[8] Sano T., Nakamura C., Yoshida T., Oyama T. (2014). Stratified mucin-producing intraepithelial lesion (SMILE) of the uterine cervix are associated with HPV integration. Pathol. Int..

[9] Lastra R.R., Park K.J., Schoolmeester J.K. (2016). Invasive stratified mucin-producing carcinoma and stratified mucin-producing intraepithelial lesion (SMILE): 15 cases presenting a spectrum of cervical neoplasia with description of a distinctive variant of invasive adenocarcinoma. Am. J. Surg. Pathol..

[10] Fukui S., Nagasaka K., Iimura N., Kanda R., Ichinose T., Sugihara T., Hiraike H., Nakagawa S., Sasajima Y., Ayabe T. (2019). Detection of HPV RNA molecules in stratified mucin-producing intraepithelial lesion (SMILE) with concurrent cervical intraepithelial lesion: A case report. Virol. J..

[11] Smedts F., Ramaekers F., Link M., Lauerova L., Troyanovsky S., Schijf C., Vooijs G.P. (1994). Detection of keratin subtypes in routinely processed cervical tissue: Implications for tumour classification and the study of cervix cancer aetiology. Virchows Arch..

[12] Lee H., Lee H., Cho K.Y. (2017). Cytokeratin7 and cytokeratin19 expression in high grade cervical intraepithelial neoplasm and squamous cell carcinoma and their possible association in cervical carcinogenesis. Diagn. Pathol..

[13] Herfs M., Yamamoto Y., Laury A., Wang X., Nucci M., McLaughlin-Drubin M.E., Münger K., Feldman S., McKeon F.D., Xian W. (2010). A discrete population of squamocolumnar junction cells implicated in the pathogenesis of cervical cancer. Proc. Natl. Acad. Sci. USA.

[14] Mirkovic J., Howitt B.E., Roncarati P., Demoulin S., Suarez-Carmona M., Hubert P., McKeon F.D., Xian W., Li A., Delvenne P. (2015). Carcinogenic HPV infection in the cervical squamo-columnar junction. J. Pathol..

[15] Kurita T., Cunha G.R., Robboy S.J., Mills A.A., Medina R.T. (2005). Differential expression of p63 isoforms in female reproductive organs. Mech. Dev..

[16] Houghton O., McCluggage W.G. (2009). The expression and diagnostic utility of p63 in the female genital tract. Adv. Anatom. Pathol..

[17] Bishop J.A., Teruya-Feldstein J., Westra W.H., Pelosi G., Travis W.D., Rekhtman N. (2012). p40 (ΔNp63) is superior to p63 for the diagnosis of pulmonary squamous cell carcinoma. Mod. Pathol..

[18] Lin Z., Liu M., Li Z., Kim C., Lee E., Kim I. (2006). DeltaNp63 protein expression in uterine cervical and endometrial cancers. J. Cancer Res. Clin. Oncol..

[19] Kocjan B.J., Maver P.J., Hošnjak L., Zidar N., Odar K., Gale N., Poljak M. (2012). Comparative evaluation of the Abbott RealTime High-Risk HPV test and INNO-LiPA HPV Genotyping Extra test for detecting and identifying human papillomaviruses in archival tissue specimens of head and neck cancers. Acta Dermatovenerol. Alp. Pannonica Adriat..

[20] Ivanuš U., Florjančič M., Jerman T. (2019). Poročilo o Rezultatih Programa ZORA v letu 2018 in Načrti za Prihodnost, Zbornik Predavanj, 9.izobraževalni dan Programa ZORA. https://zora.onko-i.si/fileadmin/user_upload/dokumenti/izobrazevanja/9ZD/zbornik_ZD/1._Urska_Ivanus_Porocilo_o_rezultatih_ZORA_-_Copy.pdf.

[21] Stolnicu S., Barsan I., Hoang L., Patel P., Terinte C., Pesci A., Aviel-Ronen S., Kiyokawa T., Alvarado-Cabrero I., Pike M.C. (2018). International endocervical adenocarcinoma criteria and classification (IECC): A new pathogenetic classification for invasive adenocarcinomas of the endocervix. Am. J. Surg. Pathol..

[22] Quint W., Jenkins D., Molijn A., Struijk L., van de Sandt M., Doorbar J., Mols J., Van Hoof C., Hardt K., Struyf F. (2012). One virus, one lesion–individual components of CIN lesions contain a specific HPV type. J. Pathol..

[23] Darragh T.M., Colgan T.J., Cox J.T., Heller D.S., Henry M.R., Luff R.D., McCalmont T., Nayar R., Palefsky J.M., Stoler M.H. (2012). The lower anogenital squamous terminology standardization project for HPV-associated lesions: Background and consensus recommendations from the College of American Pathologists and the American Society for Colposcopy and Cervical Pathology. Arch. Pathol. Lab. Med..

[24] Yatabe Y., Dacic S., Borczuk A.C., Warth A., Russell P.A., Lantuejoul S., Beasley M.B., Thunnissen E., Pelosi G., Rekhtman N. (2019). Best practices recommendations for diagnostic immunohistochemistry in lung cancer. J. Thorac. Oncol..

